# User Perceptions & Subjective Memory Concerns are Associated With Brief, Remote Cognitive Assessment Performance

**DOI:** 10.1093/geroni/igab046.2408

**Published:** 2021-12-17

**Authors:** Samuel Lee, Katherine Dorociak, Nora Mattek, Sarah Gothard, Jonathan Lee, Jeffrey Kaye, Adriana Hughes

**Affiliations:** 1 University of Minnesota, Twin Cities, Minneapolis, Minnesota, United States; 2 Palo Alto VA Health Care System, Palo Alto, California, United States; 3 Oregon Health & Science University, Portland, Oregon, United States; 4 Oregon Center for Aging and Technology, Portland, Oregon, United States; 5 Minneapolis VA Health Care System, Minneapolis, Minnesota, United States

## Abstract

Online cognitive tests offer a cost-effective, accessible means of cognitive screening and may prove especially important for individuals with memory complaints, a risk factor for cognitive impairment (Kaup et al., 2015). Although older adults’ perceptions of everyday technologies impact their uptake and adoption, there is limited understanding about how perceptions of online cognitive screening tests impact test performance. The purpose of the current study was to examine relationships between performance on a brief, self-administered, web-based cognitive assessment tool (SMART) and user perceptions (e.g., ratings of challenge and length), technology confidence, brain health activities, and memory complaints. Participants were 1336 adults without a diagnosis of cognitive impairment (Mage=60.48 years, SD=15.18; 65.8% female; 81.8% White; 21.2% with subjective memory complaints). Most participants (97%) were willing to complete the SMART again, with over half (53.5%) willing to complete the SMART on at least a weekly basis. After adjusting for age and education, better SMART performance (i.e., faster completion time) was associated with user ratings of greater ease of completion, higher technology confidence, and greater participation in brain health activities (p<.05). In a subsample aged 60+, individuals with memory complaints took longer to complete certain SMART tasks (Trail Making Test B, Total SMART) than those without memory complaints (p<.05). Results suggest that the SMART is a well-accepted tool for frequent remote cognitive screening and highlight the importance of user perceptions, technology confidence, and memory complaints on online cognitive test performance.

